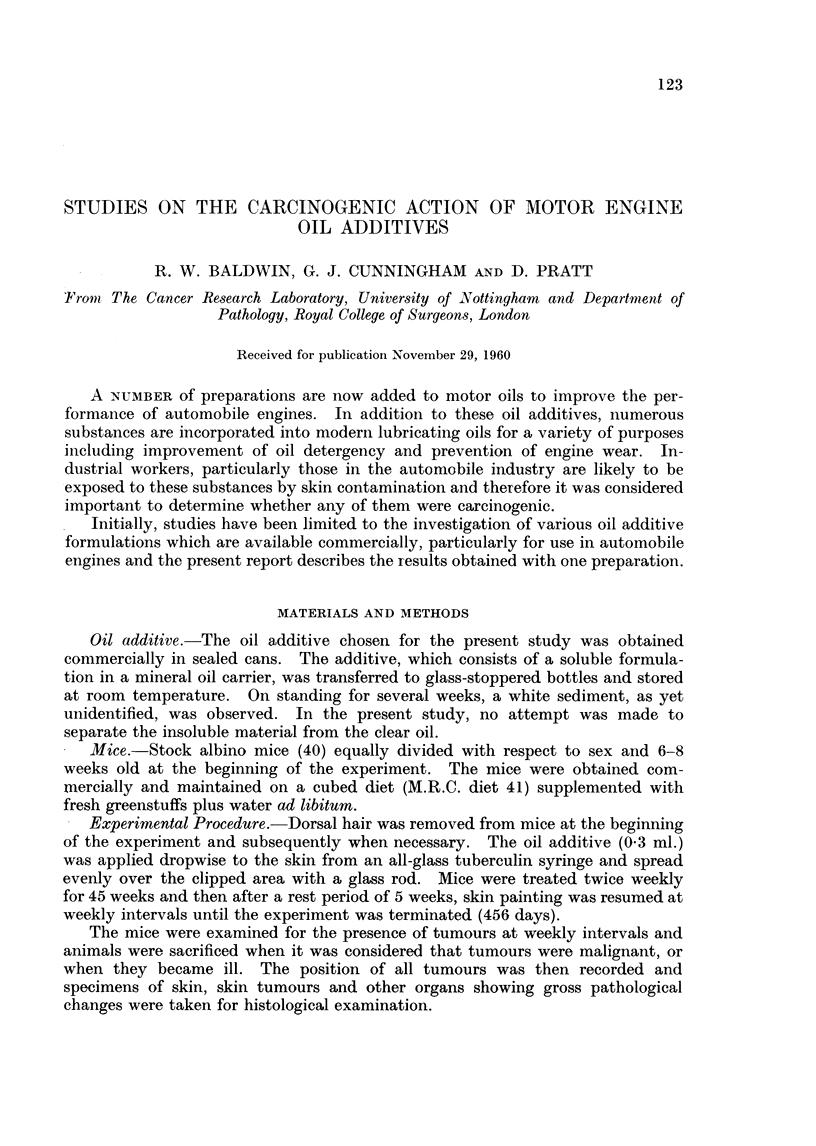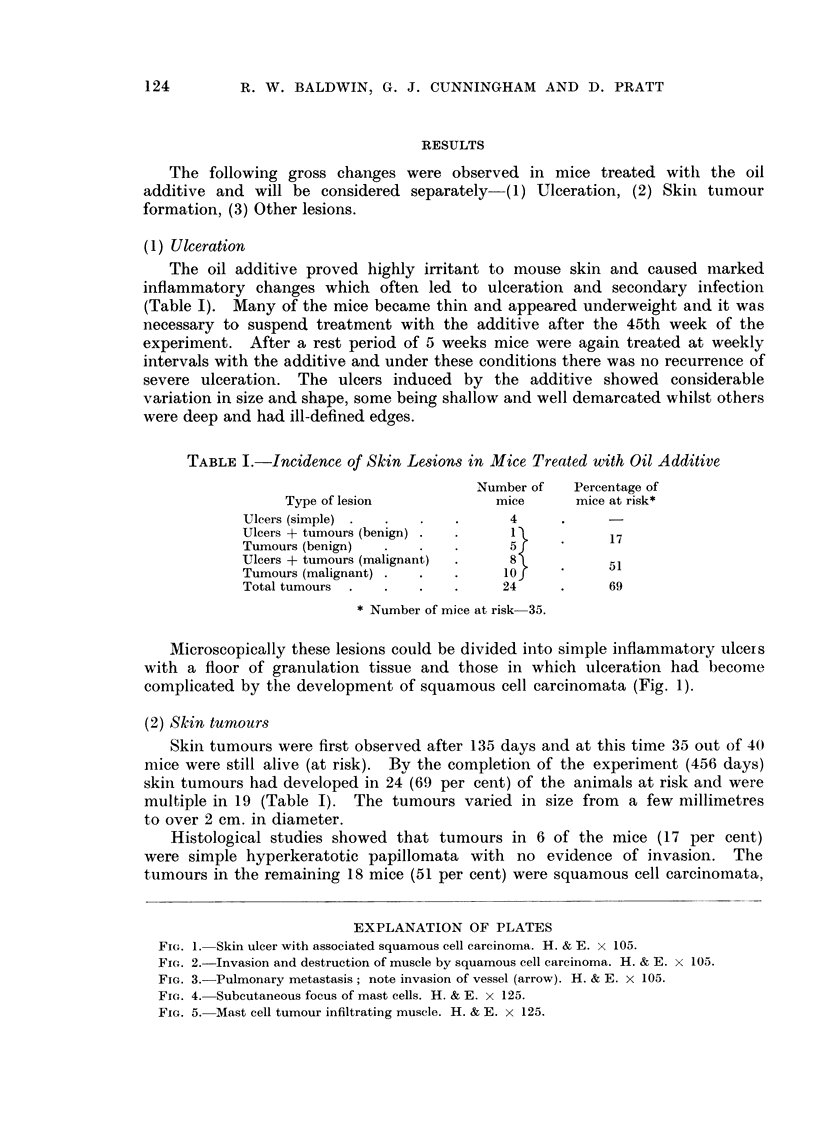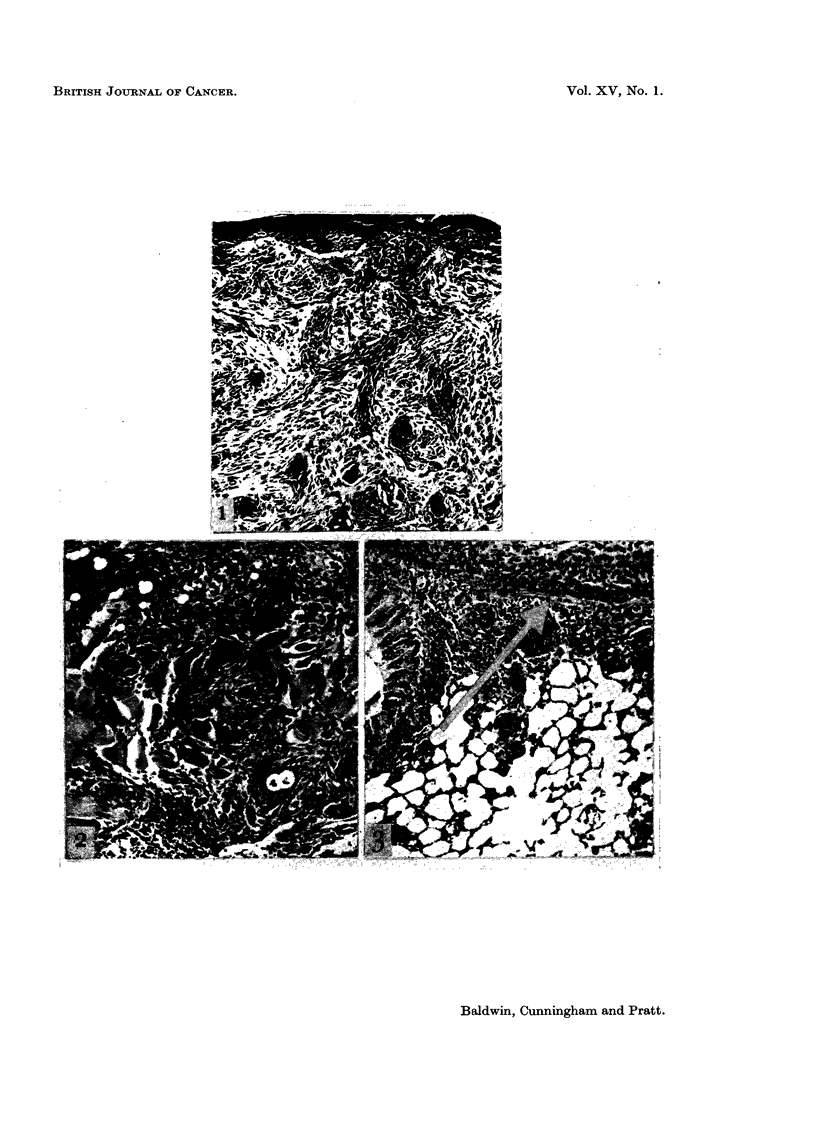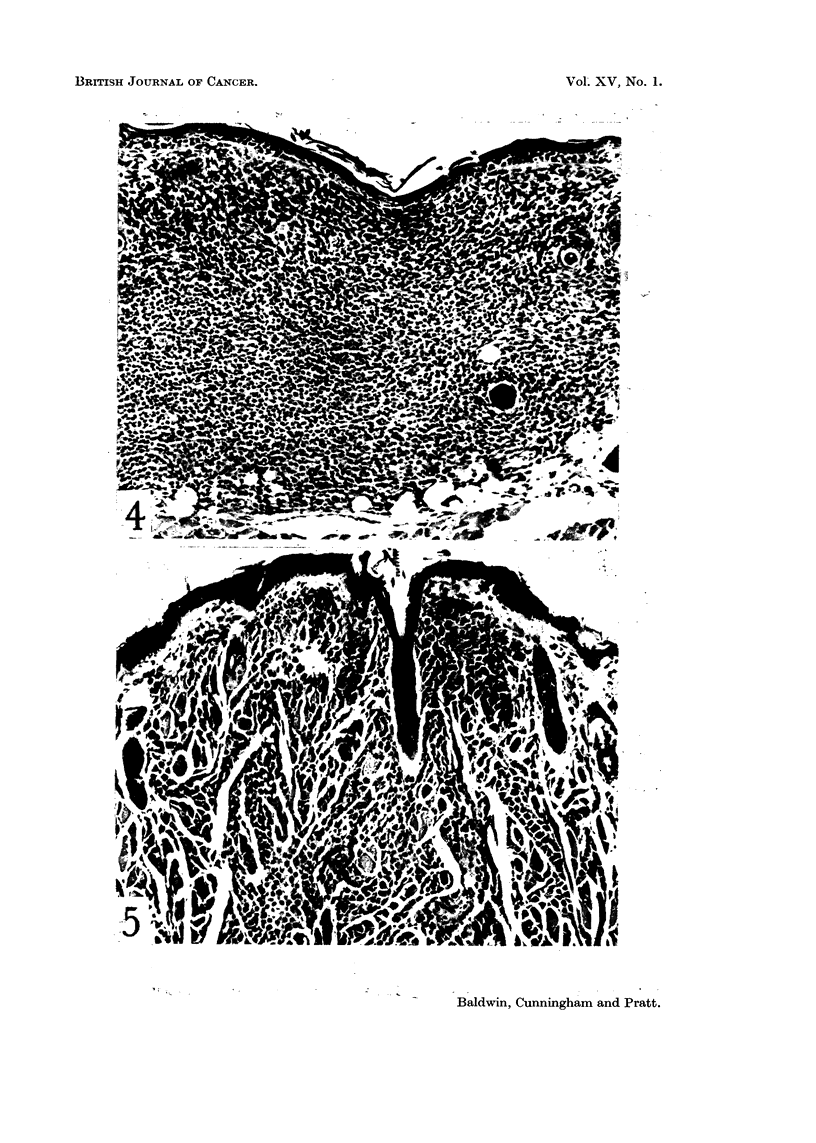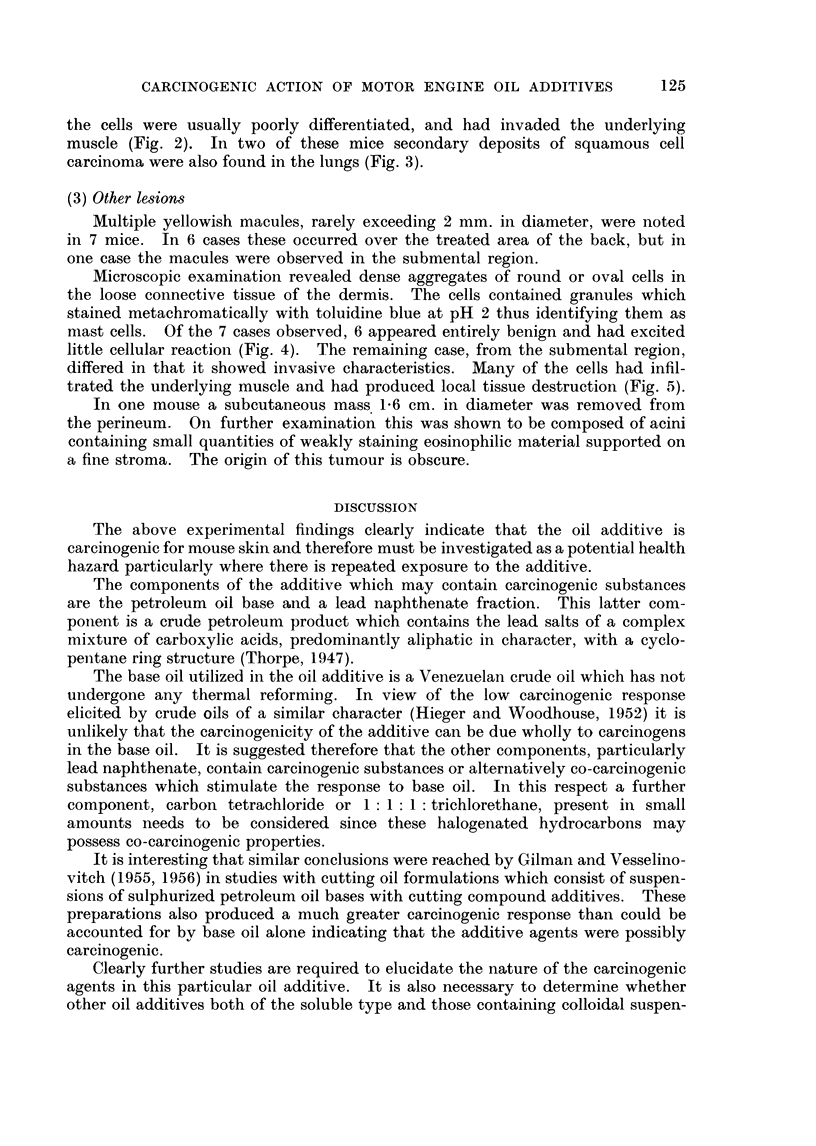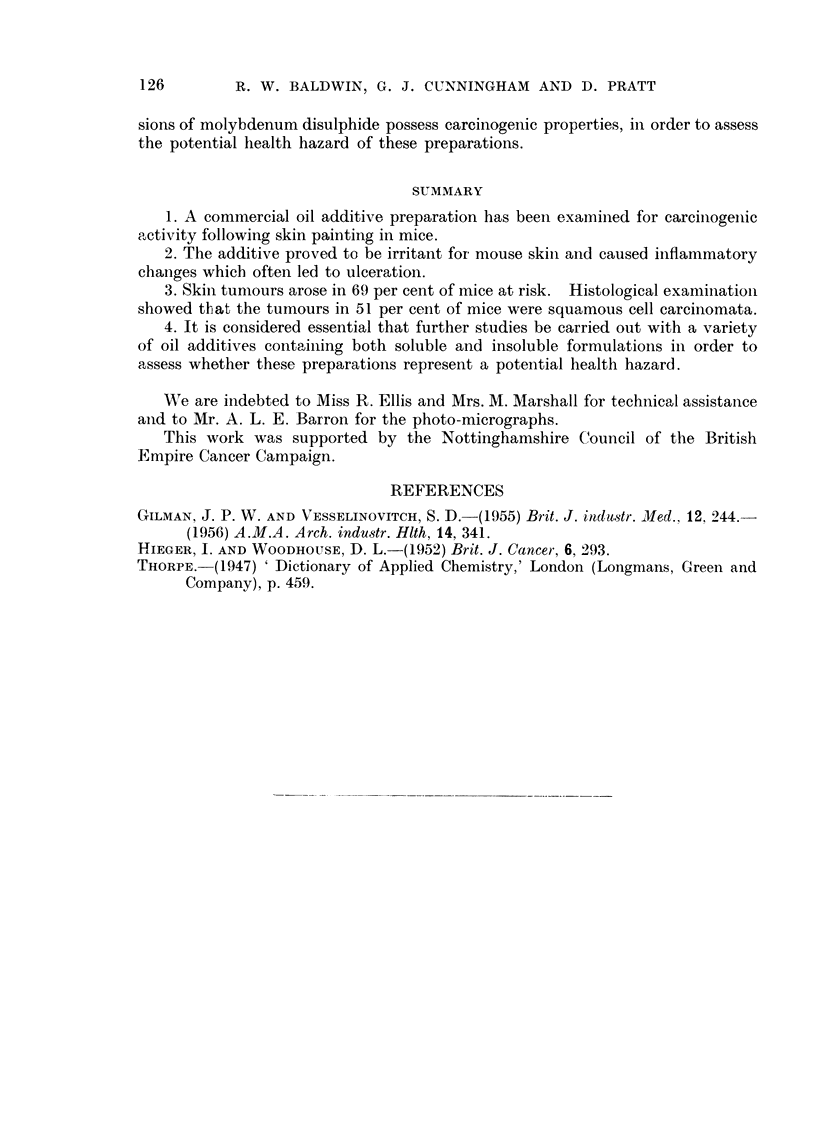# Studies on the Carcinogenic Action of Motor Engine Oil Additives

**DOI:** 10.1038/bjc.1961.14

**Published:** 1961-03

**Authors:** R. W. Baldwin, G. J. Cunningham, D. Pratt

## Abstract

**Images:**


### STUDIES ON THE CARCINOGENIC ACTION OF MOTOR ENGINE OIL ADDITIVES

R. W. BALDWIN, G. J. CUNNINGHAM AND D. PRATT

*From The Cancer Research Laboratory, University of Nottingham and Department of Pathology, Royal College of Surgeons, London*

Received for publication November 29, 1960

A NUMBER of preparations are now added to motor oils to improve the performance of automobile engines. In addition to these oil additives, numerous substances are incorporated into modern lubricating oils for a variety of purposes including improvement of oil detergency and prevention of engine wear. Industrial workers, particularly those in the automobile industry are likely to be exposed to these substances by skin contamination and therefore it was considered important to determine whether any of them were carcinogenic.

Initially, studies have been limited to the investigation of various oil additive formulations which are available commercially, particularly for use in automobile engines and the present report describes the results obtained with one preparation.

### MATERIALS AND METHODS

*Oil additive.*—The oil additive chosen for the present study was obtained commercially in sealed cans. The additive, which consists of a soluble formulation in a mineral oil carrier, was transferred to glass-stoppered bottles and stored at room temperature. On standing for several weeks, a white sediment, as yet unidentified, was observed. In the present study, no attempt was made to separate the insoluble material from the clear oil.

*Mice.*—Stock albino mice (40) equally divided with respect to sex and 6–8 weeks old at the beginning of the experiment. The mice were obtained commercially and maintained on a cubed diet (M.R.C. diet 41) supplemented with fresh greenstuffs plus water *ad libitum.*

*Experimental Procedure.*—Dorsal hair was removed from mice at the beginning of the experiment and subsequently when necessary. The oil additive (0·3 ml.) was applied dropwise to the skin from an all-glass tuberculin syringe and spread evenly over the clipped area with a glass rod. Mice were treated twice weekly for 45 weeks and then after a rest period of 5 weeks, skin painting was resumed at weekly intervals until the experiment was terminated (456 days).

The mice were examined for the presence of tumours at weekly intervals and animals were sacrificed when it was considered that tumours were malignant, or when they became ill. The position of all tumours was then recorded and specimens of skin, skin tumours and other organs showing gross pathological changes were taken for histological examination.

### RESULTS

The following gross changes were observed in mice treated with the oil additive and will be considered separately—(1) Ulceration, (2) Skin tumour formation, (3) Other lesions.

### (1) *Ulceration*

The oil additive proved highly irritant to mouse skin and caused marked inflammatory changes which often led to ulceration and secondary infection (Table I). Many of the mice became thin and appeared underweight and it was necessary to suspend treatment with the additive after the 45th week of the experiment. After a rest period of 5 weeks mice were again treated at weekly intervals with the additive and under these conditions there was no recurrence of severe ulceration. The ulcers induced by the additive showed considerable variation in size and shape, some being shallow and well demarcated whilst others were deep and had ill-defined edges.

TABLE I.—*Incidence of Skin Lesions in Mice Treated with Oil Additive*

| Type of lesion | Number of mice | Percentage of mice at risk* |
|---|---|---|
| Ulcers (simple) | 4 | — |
| Ulcers + tumours (benign) | 1 | 17 |
| Tumours (benign) | 5 | |
| Ulcers + tumours (malignant) | 8 | 51 |
| Tumours (malignant) | 10 | |
| Total tumours | 24 | 69 |

* Number of mice at risk—35.

Microscopically these lesions could be divided into simple inflammatory ulcers with a floor of granulation tissue and those in which ulceration had become complicated by the development of squamous cell carcinomata (Fig. 1).

### (2) *Skin tumours*

Skin tumours were first observed after 135 days and at this time 35 out of 40 mice were still alive (at risk). By the completion of the experiment (456 days) skin tumours had developed in 24 (69 per cent) of the animals at risk and were multiple in 19 (Table I). The tumours varied in size from a few millimetres to over 2 cm. in diameter.

Histological studies showed that tumours in 6 of the mice (17 per cent) were simple hyperkeratotic papillomata with no evidence of invasion. The tumours in the remaining 18 mice (51 per cent) were squamous cell carcinomata,

---

EXPLANATION OF PLATES

FIG. 1.—Skin ulcer with associated squamous cell carcinoma. H. & E. × 105.

FIG. 2.—Invasion and destruction of muscle by squamous cell carcinoma. H. & E. × 105.

FIG. 3.—Pulmonary metastasis ; note invasion of vessel (arrow). H. & E. × 105.

FIG. 4.—Subcutaneous focus of mast cells. H. & E. × 125.

FIG. 5.—Mast cell tumour infiltrating muscle. H. & E. × 125.

Baldwin, Cunningham and Pratt.

4

5

Baldwin, Cunningham and Pratt.

the cells were usually poorly differentiated, and had invaded the underlying muscle (Fig. 2). In two of these mice secondary deposits of squamous cell carcinoma were also found in the lungs (Fig. 3).

(3) *Other lesions*

Multiple yellowish macules, rarely exceeding 2 mm. in diameter, were noted in 7 mice. In 6 cases these occurred over the treated area of the back, but in one case the macules were observed in the submental region.

Microscopic examination revealed dense aggregates of round or oval cells in the loose connective tissue of the dermis. The cells contained granules which stained metachromatically with toluidine blue at pH 2 thus identifying them as mast cells. Of the 7 cases observed, 6 appeared entirely benign and had excited little cellular reaction (Fig. 4). The remaining case, from the submental region, differed in that it showed invasive characteristics. Many of the cells had infiltrated the underlying muscle and had produced local tissue destruction (Fig. 5).

In one mouse a subcutaneous mass 1·6 cm. in diameter was removed from the perineum. On further examination this was shown to be composed of acini containing small quantities of weakly staining eosinophilic material supported on a fine stroma. The origin of this tumour is obscure.

### DISCUSSION

The above experimental findings clearly indicate that the oil additive is carcinogenic for mouse skin and therefore must be investigated as a potential health hazard particularly where there is repeated exposure to the additive.

The components of the additive which may contain carcinogenic substances are the petroleum oil base and a lead naphthenate fraction. This latter component is a crude petroleum product which contains the lead salts of a complex mixture of carboxylic acids, predominantly aliphatic in character, with a cyclopentane ring structure (Thorpe, 1947).

The base oil utilized in the oil additive is a Venezuelan crude oil which has not undergone any thermal reforming. In view of the low carcinogenic response elicited by crude oils of a similar character (Hieger and Woodhouse, 1952) it is unlikely that the carcinogenicity of the additive can be due wholly to carcinogens in the base oil. It is suggested therefore that the other components, particularly lead naphthenate, contain carcinogenic substances or alternatively co-carcinogenic substances which stimulate the response to base oil. In this respect a further component, carbon tetrachloride or 1 : 1 : 1 : trichlorethane, present in small amounts needs to be considered since these halogenated hydrocarbons may possess co-carcinogenic properties.

It is interesting that similar conclusions were reached by Gilman and Vesselinovitch (1955, 1956) in studies with cutting oil formulations which consist of suspensions of sulphurized petroleum oil bases with cutting compound additives. These preparations also produced a much greater carcinogenic response than could be accounted for by base oil alone indicating that the additive agents were possibly carcinogenic.

Clearly further studies are required to elucidate the nature of the carcinogenic agents in this particular oil additive. It is also necessary to determine whether other oil additives both of the soluble type and those containing colloidal suspensions of molybdenum disulphide possess carcinogenic properties, in order to assess the potential health hazard of these preparations.

### SUMMARY

1. A commercial oil additive preparation has been examined for carcinogenic activity following skin painting in mice.

2. The additive proved to be irritant for mouse skin and caused inflammatory changes which often led to ulceration.

3. Skin tumours arose in 69 per cent of mice at risk. Histological examination showed that the tumours in 51 per cent of mice were squamous cell carcinomata.

4. It is considered essential that further studies be carried out with a variety of oil additives containing both soluble and insoluble formulations in order to assess whether these preparations represent a potential health hazard.

We are indebted to Miss R. Ellis and Mrs. M. Marshall for technical assistance and to Mr. A. L. E. Barron for the photo-micrographs.

This work was supported by the Nottinghamshire Council of the British Empire Cancer Campaign.

### REFERENCES

GILMAN, J. P. W. AND VESSELINOVITCH, S. D.—(1955) *Brit. J. industr. Med.*, **12**, 244.—(1956) *A.M.A. Arch. industr. Hlth*, **14**, 341.

HIEGER, I. AND WOODHOUSE, D. L.—(1952) *Brit. J. Cancer*, **6**, 293.

THORPE.—(1947) ' Dictionary of Applied Chemistry,' London (Longmans, Green and Company), p. 459.